# Effect of Tooth Abutment Height on Retention of CAD/CAM Monolithic Zirconia Crowns Cemented With Two Types of Cement: An In Vitro Study

**DOI:** 10.1155/ijod/4694705

**Published:** 2026-01-06

**Authors:** Fatemeh Soleimani, Azam Sadat Mostafavi, Hamid Jalali

**Affiliations:** ^1^ Tehran University of Medical Sciences, School of Dental Medicine, Tehran, Tehran Province, Iran, tums.ac.ir

**Keywords:** dental abutments, dental prosthesis retention, resin cements, TheraCem

## Abstract

**Statement of the Problem:**

Retention is still a primary concern in conservatively managing short clinical crowns (SCC) and minimal restorative space. In modern dentistry, there is a growing expectation for the durability and retention of indirect restorations, along with a high esthetic demand, even in unfavorable underlying conditions.

**Purpose:**

This study aims to establish a reliable approach for the conservative treatment of SCCs using monolithic zirconia crowns (MZC).

**Materials and Methods:**

Sixty human molars were collected and mounted in resin blocks. The teeth were prepared with two different abutment heights (2 and 4 mm). MZCs with buccal and lingual projections were designed and milled after digital scanning. Half of the restorations were cemented with TheraCem (TC) calcium silicate–based self‐adhesive resin cement, while the remaining half were cemented with glass ionomer (GI) cement. The specimens underwent thermocycling and cyclic loading. A traction test was then performed in the universal testing machine to evaluate the debonding forces. Data was analyzed using Pearson correlation and one‐way ANOVA tests.

**Results:**

A strong positive correlation was shown in Pearson analysis between the assigned group and the pull‐out force (*r =* 0.95, *p*‐value ≤ 0.01). In addition, ANOVA found a significant mean difference in the pairwise comparison of groups. Increasing abutment height in groups with either GI or TC cement type resulted in increased measured force. More importantly, TC groups have shown a greater average value than specimens with GI cement.

**Conclusions:**

We demonstrated the significant influence of TC cement on the pull‐out force measurement, outperforming the GI cement. The abutment height also showed a smaller contribution to the force measurement. Low‐thickness monolithic zirconia restorations cemented with self‐adhesive resin cement can be recommended as a reliable and effective treatment option for clinicians managing cases with SCCs and compromised retention.

## 1. Introduction

In modern dentistry, restorations aim to address esthetic, phonetic, biological, and functional requirements, as well as ensure adequate retention and resistance [1]. Studies have found that the average longevity of a full‐coverage restoration is between 10 and 15 years [2, 3]. Likewise, the common causes of their failure include lack of retention and recurrent caries [4, 5]. Retention is a key factor in the clinical success of indirect restorative techniques [6, 7]. Based on the glossary of prosthodontic terms, retention refers to the prevention of restoration dislodgement in the direction of insertion [8]. Crown retention depends on the geometrical configuration of the tooth preparation, such as the apical‐coronal height of the abutment stump, total occlusal convergence (TOC) angle, surface area, and surface roughness [9, 10]. Goodacre recommends a TOC range of 10° to 20° and proposes a preparation height of 4.0 mm as the minimal occlusal‐cervical dimension for prepared molars [11].

A short clinical crown (SCC) is described as any tooth having less than 2 mm of intact, opposing parallel walls remaining after occlusal and axial reduction [12]. Common leading causes of SCC include caries, erosion, attrition, trauma, excessive tooth preparation, eruption disharmony, and congenital or genetic tooth malformations such as amelogenesis imperfecta [13, 14]. When restoring an SCC, the clinician may attempt to increase preparation height by placing a subgingival margin, performing surgical crown lengthening, utilizing orthodontic eruption, altering tooth reduction design, and applying foundation restorations. Nonconservative methods encroach upon the biological width, jeopardize the periodontal attachment, and are, therefore, undesirable [12, 15–17].

Specifically, prosthetic options for fixed restoration are casting gold, lithium disilicate, porcelain fuzed to metal (PFM), porcelain fused to zirconium (PFZ), and monolithic zirconia crown (MZC) [18, 19]. A comparison between restorative materials showed that the retention value of zirconia copings was higher than that of cast metal copings. This may be attributed to the superior fit accuracy of CAD/CAM‐fabricated zirconia copings compared to cast metal copings utilizing the lost‐wax technique [20–22].

Today, zirconia is considered a suitable restorative material for fixed prosthetic rehabilitation due to its optical properties, high biocompatibility, shade stability, low heat conductivity, and favorable physical strength, as it can withstand bite forces of over 150 N in the molar region [23–26]. PFZ and PFM restorations are at risk of porcelain debonding and/or chipping [27, 28]. In contrast, the monolithic zirconia blank is multilayered and precolored, eliminating porcelain layering and allowing for the fabrication of MZCs with enhanced esthetics and long‐term durability [29–31].

Given the limited restorative space, monolithic zirconia’s conservative preparation requirements, and high strength to resist fracture with a minimum thickness of 0.5 mm would be a desirable feature [32, 33]. These favorable characteristics, along with reduced wear on the antagonist teeth and minimal tooth preparation, enhance conservative tooth preparation and improve crown retention despite inadequate abutment height [34–36].

Desirable retention is a critical concern in SCCs; therefore, a secondary retention form and the use of adhesive luting agents ought to be considered [37]. A strong chemo‐mechanical bond to zirconia ceramic is obtained by using laser irradiation or airborne particle abrasion, followed by bonding to metal oxides of zirconia through a resin cement containing the monomer 10‐methacryloyloxydecyl dihydrogen phosphate (10‐MDP) [38–40]. Airborne particle abrasion is essential for increasing surface irregularities, enhancing the chemical bond strength between resin cement and highly crystallized zirconia‐reinforced inert ceramics [40]. Laser treatment enhances the shear bond strength of presintered zirconia to resin cement more effectively than air abrasion [41]. Retentive cavities are most likely not mandatory [42].

10‐MDP monomers contain a functional phosphate ester group that directly forms a chemical bond with metal oxides of zirconia. The reliable bonding of adhesive resin cement to zirconia crown improves the marginal seal, retention, and subsequently the fracture resistance of the restoration [43]. A 10‐MDP‐containing primer, such as Z‐Primer, can be applied to the zirconia surface to reduce the contact angle, decrease surface hydrophobicity, and enhance bonding strength [44].

We often encounter patients with SCCs and limited restorative space in daily practice. Treatment of such cases is clinically challenging, especially when anatomical constraints (e.g., short roots), medical conditions, or patient preferences rule out surgical crown‐lengthening or orthodontic extrusion. As a result, clinicians are often forced to rely on conservative, prosthetic‐based solutions to achieve functional and esthetic outcomes. This challenge is particularly evident in patients with amelogenesis imperfecta, a hereditary condition characterized by enamel defects, tooth sensitivity, and significantly reduced crown height—conditions that commonly require full coverage restorations for rehabilitation [45]. Despite the clinical significance, there is currently no universally accepted gold standard for restoring SCCs—especially in patients with underlying enamel defects like amelogenesis imperfecta—or for selecting the most effective cementation protocol in such cases. Moreover, the effectiveness of MZCs in cases with limited abutment height remains an area of ongoing investigation.

Our study is novel in that it evaluates and compares the retentive performance of glass ionomer (GI) and TheraCem (TC) resin cements in different abutment heights using human teeth. Notably, we focus on the potential of TC resin cement to enhance retention in compromised clinical scenarios, offering valuable insights for more predictable and conservative treatment planning in patients where traditional surgical or orthodontic options are not viable. These findings may inform clinical decision‐making in prosthodontics, particularly when managing complex conditions such as amelogenesis imperfecta or other cases involving (SCCs).

## 2. Materials and Methods

**Figure 1:**
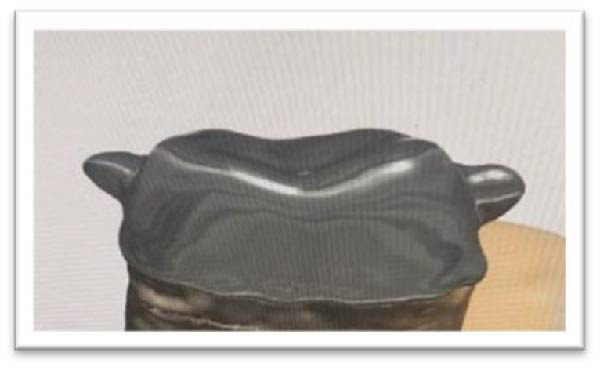
This image demonstrates the designation of wings for the pull‐out test.

The present in vitro study utilized 60 human mandibular molars of comparable size, extracted within 2 months due to periodontal or orthodontic indications. Informed consent was obtained from all patients, following the protocols of the clinical research ethics board. The inclusion criteria for recruiting subjects were caries‐free teeth with neither cracks nor attrition. After extraction, blood contamination was cleaned, and teeth were immersed in 0.5% sodium hypochlorite for 5 min. Then, they were stored in tap water at 4°C, with a weekly water replacement. To standardize sample size, buccolingual and mesiodistal dimensions were measured using a digital caliper (ERSTE QUALITAT, Germany) with 0.01 mm accuracy. Only teeth with a diameter of 10 mm ± 1 mm were included in our study. The root surfaces were coated with a thin layer of wax, positioned in a polyvinyl chloride (PVC) matrix, and stabilized with auto‐polymerizing resin 2 mm below the buccal cementoenamel junction. The teeth were randomly divided into four groups (each group with 15 samples) and prepared for full‐coverage ceramic crowns. The occlusal surfaces were prepared with a barrel‐shaped burr (ISO 811L, Jota, Switzerland) to expose dentin, using a surveyor to standardize a perpendicular orientation. In our investigations, all preparations were conducted using a high‐speed handpiece attached to a surveyor, following the method introduced by Ernst et al. [46]. Axial surfaces were prepared with a round‐end conical diamond bur (ISO 852.016, Jota, Switzerland) to produce a 6° taper and 0.5 mm finishing line at varying distances 2 mm (SCC) and 4 mm from the proximal marginal ridges, according to group assignment. Occlusal convergence was verified via photographs and Adobe Photoshop CS2 software (Version 9.0, Adobe Systems Inc., San Jose, CA, USA). Each tooth was scanned with an intraoral scanner (TRIOS4, 3Shape, Copenhagen, Denmark), and CAD files were transferred to Exocad software (GmbH, Darmstadt, Germany) for crown design. MZCs (inCoris TZIC, SIRONA, Germany) were fabricated using CAD/CAM technology with a cement spacer of 40 µm, positioned 0.5 mm from the finishing line and with uniform thickness across the axial and occlusal surfaces. Figure 1 illustrates a two‐wing creation on the proximal crown surfaces to facilitate retention during the pull‐out test.

The crowns were adjusted on each tooth using a disclosing silicone material (Fit Checker, GC Co. Alsip, IL, USA). Internal surfaces were abraded using 50 *μ*m aluminum oxide particles at 1.5 bar pressure for 15 s and then cleaned in an ultrasonic bath with isopropyl alcohol (MOEHAIR, USA) for 3 min. TC (BISCO, Japan) specimens received Z‐Primer (Z‐Prime Plus, BISCO, IL, USA) on the intaglio surface, air‐dried for 5 s, and TC cement was applied uniformly inside the crown. Crowns were seated using a 5 kg weight for 2 min and light‐cured for 25 s per side using a Coltolux LED (Coltène/Whaledent Inc., USA) at 600 mW/cm^2^. A consistent seating pressure was applied during crown cementation using a 5 kg weight, as the pressure can influence the crown seating, internal adaptation, and final strength of cement [47, 48]. For the GI (GC, Japan) specimens, cement was mixed for 20 s at 24°C, applied, and seated with the same weight and duration.

The specimens were removed from the PVC matrix and stored in distilled water at 37°C (pH 7) for 24 h, then thermocycled between 5°C and 55°C for 1500 cycles (60 s each) [49]. The roots were serrated to enhance retention in acrylic blocks, and dye was applied. To simulate the periodontal ligament (PDL), a thin layer of dye was applied to the root surface. This approach replicates the biomechanical behavior of the PDL and minimizes the risk of tooth extraction during the pull‐out test [50]. Specimens were mounted in acrylic blocks (20 × 20 × 30 mm) with a 1‐mm apical margin from cementoenamel junction to simulate clinical conditions, then subjected to cyclic loading at a 30° angle for 500,000 cycles at 80 N (2 Hz). The maximum loading angle of 30° was selected for this study [51].

**Figure 2:**
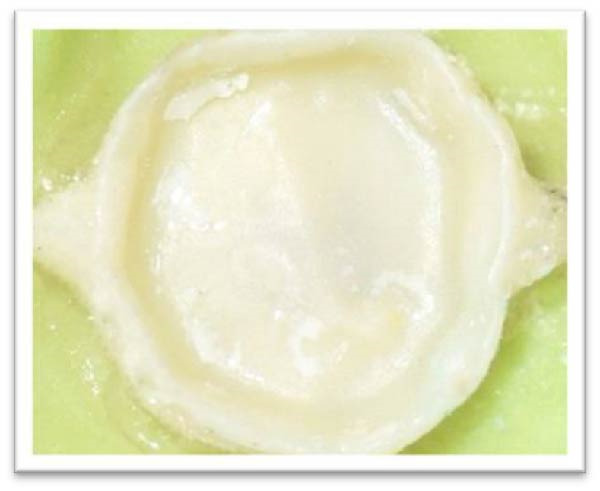
Failure mode evaluation and classification.

The pull‐out test was conducted using a universal testing machine (Zwick/Roell Z050, Ulm, Germany) at 1 mm/min crosshead speed, with retention force measured in Newtons(N). Finally, the failure mode was determined using a magnifier (Zeiss Optics; D40, 10x, Carl Zeiss, Oberkochen, Germany). It was classified into three categories: adhesive failure of crown intaglio and cement (cement remains on the tooth), adhesive failure of tooth and cement (cement remains on crown intaglio), and mixed adhesive failure of cement remaining on both crown and tooth. Figure 2 demonstrates the consistency of the study by controlling confounding factors such as cement thickness, zirconia surface treatment, and tooth preparation variables such as taper, roughness, and undercuts.

### 2.1. Statistical Analysis

This study investigated the influences of abutment height and cement type on the pull‐out force. The dataset was analyzed to compare pull‐out force among four groups categorized by cement type (GI and TC) and abutment height (2 mm [SCC] and 4 mm). Descriptive statistics, including mean and standard deviation (SD) of force, were measured for each group. Pearson’s correlation analysis was conducted to evaluate the linear relationship between the assigned groups and force. The Kolmogorov–Smirnov test was used to assess the normality of the dataset. A one‐way ANOVA was performed for normally distributed force measurements, followed by Tukey’s post hoc test. Lastly, pairwise comparisons were conducted using Tukey’s HSD test results to identify statistically significant group differences. The level of statistical significance was set at *p*  < 0.05 for all analyses. SPSS (IBM, Armonk, NY, USA) version 28.0 was used for all statistical analyses and graph generation.

## 3. Results

A total of 60 specimens were included and randomly assigned to four experimental groups. Table 1 presents the mean pull‐out force values and standard deviations for each group. A strong positive correlation was observed between abutment height and retention force, as shown in the Pearson correlation analysis (*r* = 0.95, *p*  < 0.05) in Figure 3A.

Figure 3The scatter plot (A) demonstrates the strong positive Pearson correlation from group 1 to group 4. A pairwise comparison of groups (B) with box plots is shown based on the ANOVA test result. Statistically significant differences were noted in the mean value of the pull‐out force among all comparisons. Group 1: GI, 2 mm; Group 2: GI, 4 mm; Group 3: TheraCem, 2 mm; Group 4: TheraCem, 4 mm.

**Figure:**
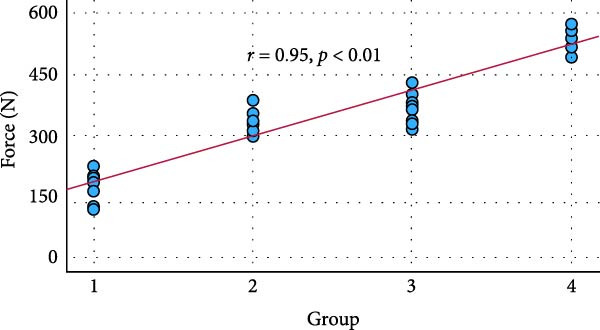
(A)

**Figure:**
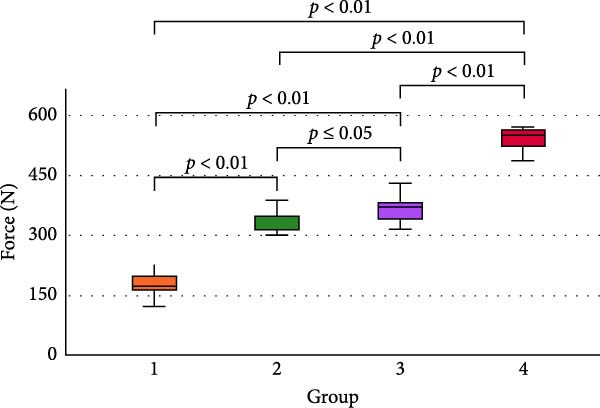
(B)

**Table 1:** This table demonstrates the effect of increasing abutment height on the pull‐out force in each cement type group.

Group	Cement type	Abutment height (mm)	Pull‐out force (Newton)
1 (GI2)	GI	2	179 ± 30
2 (GI4)	GI	4	332 ± 23
3 (TC2)	TheraCem	2	367 ± 30
4 (TC4)	TheraCem	4	541 ± 25

*Note:* In addition, groups with TheraCem cement are showing higher force mean values than groups with GI cement type.

Data met the assumptions of normality, and a one‐way ANOVA revealed statistically significant differences among groups (*p*  < 0.001). Figure 3B illustrates the results of the post hoc Tukey’s HSD test, which confirmed significant differences in pull‐out forces across all pairwise group comparisons.

Failure mode analysis revealed that adhesive failure was predominant. As shown in Table 2, all GI cement specimens exhibited residual cement on the tooth surface, indicating failure at the crown‐cement interface. In contrast, 70% of the TC group displayed cement remnants within the crown, while 30% showed a mixed adhesive failure pattern.

**Table 2:** Remaining cement attachment location.

Groups	Tooth	Crown intaglio	Both
GI	30	0	0
TheraCem	0	21	9

*Note:* While GI Cement remains were observed on the tooth, the TheraCem cement remains were attached to both the crown intaglio and the tooth.

## 4. Discussion

Our findings indicate that a combination of the cement type and the abutment height is a critical factor in retaining full‐coverage restorations. TC cement shows a strong positive effect on the pull‐out force compared to GI cement. This suggests that TC resin cement provides superior retention, which could be attributed to its enhanced bonding capabilities. However, abutment height still leads to a smaller but positive increase in the force among each cement group. The increased surface area for cement bonding, associated with a higher abutment, provides more anchorage, which translates to higher retention values. The positive correlation coefficient verifies the relationship between abutment height, cement type, and retention.

It has been shown that among the factors influencing crown dislodgement force, the most important ones are a combination of mechanical and chemical methods, like abutment height, convergence angle, and the luting agent [52, 53].

The study by Osman indicated that the pull‐off force increased nonsignificantly from 1mm to 2 mm abutment height (both are SCC), with a *p*‐value of 0.62 [54]. The mean separation force of crowns with a 3‐mm abutment height cemented with resin cement was significantly greater than that of crowns with a 5‐mm abutment height cemented with zinc phosphate. This finding aligns with the result of our study [55].

Previous studies showed that resin cement provides superior retention compared to resin‐modified GI and conventional cement. Furthermore, the retention of different resin cements to zirconia copings on prepared teeth was not statistically significant, which can be due to their similar composition of phosphate groups [56, 57].

In the present study, adhesive failure was the predominant failure mode, with GI cement typically remaining on the tooth. In contrast, TC self‐adhesive cement was more frequently observed on the crown intaglio, indicating material‐dependent performance differences. Evaluation of failure mode in the TC group revealed an adhesive failure of cement‐tooth in 70% of specimens, and 30% showed a mixed adhesive failure. Adhesive failure between human dentin and cement is the most common failure mode of self‐adhesive, self‐etching cement [58]. Ali et al. demonstrated that the adhesive resin cement remained mostly on the tooth when using cement with a dentin‐bonding system. In contrast, the cement remained primarily on the zirconia copings when using self‐adhesive resin cement [59]. These findings corroborate the failure mode observed in our study.

The self‐adhesive resin cement does not completely dissolve the smear layer, resulting in minimal to no hybrid layer formation. Therefore, it can be concluded that chemical bonding, rather than micromechanical bonding, is primarily responsible for the adhesion of resin cement to the tooth [60]. Self‐adhesive cement containing 10‐MDP acidic hydrophilic monomers initially has a low pH, allowing the acidic groups to form a stable bond with the calcium hydroxyapatite through the smear layer. Alkaline particles neutralize the remaining acidic groups by releasing calcium, fluoride, sodium, and silicate ions, while calcium hydroxide accelerates the neutralization [61].

Improving the weak micromechanical interlocking requires the formation of microirregularities on the dentin surface by dissolving calcium phosphates. The resin then infiltrates these irregularities and forms a hybrid layer [62]. Self‐adhesive resin cement exhibits excellent characteristics in terms of flexural strength, modulus of elasticity, and water absorption, which are attributed to the presence of the 10‐MDP monomer [63].

In general, three feasible models are proposed as mechanisms of interaction between the phosphate functional group of 10‐MDP with zirconia: hydrogen bonding, ionic bonding, and chemical interaction [38, 40, 64, 65]. On the other hand, GI cement exhibits adhesive failure at the ceramic‐cement interface after the traction test. A weaker bond to zirconia is due to the absence of a bonding agent. The adhesion of GI cement to the tooth structure is attributed to two interrelated mechanisms: micromechanical interlocking and chemical bonding. The self‐etching polyacid components facilitate both hydrogen and ionic interactions [66–68].

Clinically acceptable retention is typically considered to range from 200 to 600 Newtons [69]. In the present study, a 2‐mm abutment height cemented with GI shows the lowest and clinically unacceptable retention. On the other hand, for a 2‐mm abutment height SCC cemented with TC, the retention value was clinically acceptable. For a 4‐mm abutment height, both cements provide clinically acceptable retention.

Furthermore, the benefits of monolithic zirconia used with TC resin cement become especially clear compared to cast gold restorations with conventional cement. This is particularly true for SCCs or cases of amelogenesis imperfecta, where it can be difficult to achieve adequate retention conservatively. To the best of our knowledge, this is the first study to investigate and compare the retention of TC and GI cement on two different abutment heights with MZCs to establish a reliable clinical implication for SCCs. A systematic review showed that there is no consensus among papers on the clinical approach to managing SCCs [70].

The present study had several limitations. First, the results were obtained in an in vitro environment, which does not fully replicate the complex conditions of the oral cavity, such as moisture control and isolation challenges that may influence cement performance. Second, our sample size was relatively small. Third, different methods of zirconia crown preparation could be evaluated to enhance retention in cases with minimal restorative space or SCCs. Finally, we did not assess variations in axial taper. Future studies would benefit from being conducted in a clinical setting to account for in vivo variables. Moreover, we recommend including samples from different tooth positions and types, along with a larger sample size, to improve the generalizability of the findings.

## 5. Conclusions

Our study demonstrates that monolithic zirconia crown with TC resin cement effectively addresses retention concerns by significantly enhancing crown retention to a clinically acceptable level in SCC.

## Conflicts of Interest

The authors declare no conflicts of interest.

## Author Contributions

Conception, design: Azam Sadat Mostafavi, Fatemeh Soleimani, Hamid Jalali.Administrative support:Hamid Jalali, Azam Sadat Mostafavi. Provision of study materials or patients:Fatemeh Soleimani, Azam Sadat Mostafavi, Hamid Jalali. Collection and assembly of data:Fatemeh Soleimani.Data analysis and interpretation: Fatemeh Soleimani, Azam Sadat Mostafavi. Manuscript writing: Fatemeh Soleimani, Azam Sadat Mostafavi.Final approval of manuscript: Fatemeh Soleimani, Azam Sadat Mostafavi.

## Funding

This work did not receive any specific grant from funding agencies in the public, commercial, or not‐for‐profit sectors.

## Data Availability

The experimental data used to support the findings of this study are available from the corresponding author upon request.
